# Improving image quality in fast, time-resolved micro-CT by weighted back projection

**DOI:** 10.1038/s41598-020-74827-x

**Published:** 2020-10-22

**Authors:** Marjolein Heyndrickx, Tom Bultreys, Wannes Goethals, Luc Van Hoorebeke, Matthieu N. Boone

**Affiliations:** 1 UGCT/Radiation Physics, Dept. Physics and Astronomy, Ghent University, Proeftuinstraat 86 - Building N12, 9000 Ghent, Belgium; 2 UGCT / PProgRess, Dept. Geology, Ghent University, Krijgslaan 281 - Building S8, 9000 Ghent, Belgium

**Keywords:** Imaging techniques, Design, synthesis and processing, Software

## Abstract

Time-resolved micro-CT is an increasingly powerful technique for studying dynamic processes in materials and structures. However, it is still difficult to study very fast processes with this technique, since fast scanning is typically associated with high image noise levels. We present weighted back projection, a technique applicable in iterative reconstruction methods using two types of prior knowledge: (1) a virtual starting volume resembling the sample, for example obtained from a scan before the dynamic process was initiated, and (2) knowledge on which regions in the sample are more likely to undergo the dynamic process. Therefore, processes on which this technique is applicable are preferably occurring within a static grid. Weighted back projection has the ability to handle small errors in the prior knowledge, while similar 4D micro-CT techniques require the prior knowledge to be exactly correct. It incorporates the prior knowledge within the reconstruction by using a weight volume, representing for each voxel its probability of undergoing the dynamic process. Qualitative analysis on a sparse subset of projection data from a real micro-CT experiment indicates that this method requires significantly fewer projection angles to converge to a correct volume. This can lead to an improved temporal resolution.

## Introduction

High resolution X-ray CT or $\mu$CT^1^ is a technique used to obtain virtual 3-dimensional representations ofobjects, materials and structures, including both the internal and external features and analyse these^2–4^. Similar to medical CT, a $\mu$CT system uses an X-ray source, emitting X-rays towards the sample, and an X-ray detector recording the radiographs, i.e. the spatial distribution of the intensity of the X-ray beam after passing through the sample. By rotating either the sample or the gantry (holding the source and detector), radiographs from different angles are recorded. After collecting the radiographs from a sufficient angular range, a reconstruction algorithm calculates a virtual 3-dimensional volume representing the local linear attenuation coefficients within the sample from these radiographs.

Many research disciplines require the ability to visualise and analyse dynamic 3D processes in situ, exploiting the non-destructive nature of X-ray CT imaging. These processes span a very wide range of applications and origins. Biological systems such as a blowfly can be investigated to study relatively slow processes such as pupation^5^ as well as very fast processes such as wingbeat^6^, requiring gating to achieve the acquisition speed^7^. Already since the early days of high-resolution X-ray imaging, foam-like structures have frequently been the subject of imaging studies due to the relative simplicity of inducing the relevant processes^8–10^. With the development of advanced add-on equipment such laser furnaces^11^ and flow cells^12^, it has become possible to investigate processes in-situ in natural materials, e.g. two-phase fluid flow in geomaterials^13,14^, cracking and (self-)healing in engineered materials^15,16^ and many more. Recent advances in 4D imaging have led to an increased spatial resolution^17^, temporal resolution^18^ and sustainability of the acquisition^19^, and allowed for a combination of 4D with advanced X-ray imaging techniques^20^.

A fundamental issue in dynamic X-ray imaging is the motion or deformation of the object during the acquisition of the tomographic dataset. Indeed, typical tomographic reconstruction algorithms assume the object remains static during the acquisition, and this assumption is by definition violated in dynamic micro-CT or 4D-$\mu$CT. As a consequence, reconstructions of 4D-$\mu$CT scans typically suffer from motion artefacts. In order to minimise these, 4D-$\mu$CT scans are performed fast, as to minimise the acquisition time of the radiographs for a single 3D reconstruction. However, reducing the acquisition time reduces the total amount of photons recorded and thus increases the relative image noise. This complicates the analysis of these datasets.

Prior knowledge about the sample or the dynamic process has a great potential to enhance the analysis of 4D-$\mu$CT imaging, usually building on iterative reconstruction algorithms^21^. The most simple of these is the use of an initial volume^22^, in which a volume resembling the sample is known and used as a starting point for an iterative reconstruction. This knowledge can be based on design (for example for an industrial component) or be the result of a prior, high-quality $\mu$CT scan acquired before the dynamic process started. Using an initial volume can lead to faster convergence and requires less available data for similar reconstruction quality.

However, the way in which the incorporated prior knowledge influences the final resulting volume is important. If the influence is too strong, small errors in the prior knowledge could propagate to wrong results that might be hard to detect. Therefore, any algorithm must be able to robustly deal with at least a small margin of error in the prior knowledge.

In this paper, we will describe a novel technique that uses prior knowledge about which regions in the sample are static and which regions may be dynamic, i.e. knowledge on where in the sample the dynamic process may occur. This type of prior knowledge has been used in other algorithms in literature, such as the method presented by Myers *et al.*^23^ and region based 4D tomographic reconstruction^24^. In both of these, the static regions are enforced in order not to deviate from their initial value. In contrast, the proposed methodology allows for such deviations, making it more robust to small errors on the prior knowledge. In Myers’ method^23^, the prior knowledge on the static regions is supplemented with knowledge about the physics of the dynamic process and the fact that only two attenuation coefficients are possible. This is implemented as corrections to the reconstruction volumes in between the iterations of an iterative reconstruction and after the reconstruction has converged.

In region based reconstruction^24^, two separate SIRT reconstructions are performed. One, called the static reconstruction, uses the complete projection data, the other uses a small fraction around the time that is being reconstructed and is performed for each time step. Both reconstructions in this method, static and dynamic, reconstruct the entire volume, i.e. the static reconstruction also reconstructs the dynamic region and vice versa. The resulting volume for one time step uses as attenuation coefficients for the static regions the first reconstruction and as attenuation coefficients for the dynamic regions the second one. Therefore, in this technique, similarly to Myers’ method^23^, the prior knowledge of static and dynamic regions is incorporated in between iterations and at the end. In the weighted back projection method proposed here, on the other hand, the prior knowledge on static and dynamic regions is incorporated in the reconstruction algorithm itself, so it is computationally less demanding.

## Proposed method: weighted back projection

Weighted back projection is an adaptation on 3D iterative reconstruction for computed tomography. Although SART^25^ is used in this study, the proposed methodology can be implemented in any iterative reconstruction algorithm in a very similar way. In the following, weighted back projection is explained based on a 3D reconstruction algorithm, as the extension towards 4D is trivial.

In iterative reconstruction schemes, a 3D volume is reconstructed by stepwise alteration of an intermediate solution, starting from a certain starting volume until the solution converges. The alteration is based on the difference between the real (measured) transmission image(s) and the expected (simulated) image(s) of the intermediate solution at that iteration, sometimes complemented with other terms to improve stability and/or convergence speed. This difference is back projected over the complete volume, updating every voxel in the volume. Using the Kaczmarz method^26^, this update step for any voxel *j* from the contribution of pixel *i* is written as

1 $$\begin{aligned} \mu _{j}^{k+1} = \mu _{j}^{k} + \alpha \frac{p_i - q_i^k}{ \sum _m s_{im}^2} s_{ij} \end{aligned}$$

where *k* is the iteration counter, *i* is the detector pixel on which voxel *j* is projected with a relative contribution $s_{ij}$, $p_i$ and $q_i^k$ are the measured and the simulated attenuation data, respectively, and $\alpha$ is a relaxation factor ($\alpha \in [0,1]$), influencing the susceptibility to image noise and the convergence^22^. The algorithm is iterated until a specified convergence criterium is met.

However, in many dynamic processes, certain voxels have a higher probability to change during the dynamic process than others. The idea behind weighted back projection is to assign a weight $w_j$ to every voxel, corresponding to this probability. A high value is given to dynamic regions that are expected to change due to the process, whereas a low weight is given to regions which are expected to remain static. This weight is included in the back projection step of an iterative reconstruction, instead of in between iterations as in most 4D-$\mu$CT techniques, and assures most of the correction is assigned to dynamic voxels. Equation (1) is adapted as follows:

2 $$\begin{aligned} \mu _j^{k+1} = \mu _j^{k} + \alpha \frac{w_j}{W_i} \frac{p_i-q_i^k}{\sum _m s_{im}^2} s_{ij} \end{aligned}$$

in which the normalisation factor $W_i$ ensures that the same total amount $p_i-q_i^k$ is back projected over the complete ray $X_i$, compared to a back projection in a normal iterative reconstruction, where all weights are 1. It is defined by the following equation:

3 $$\begin{aligned} W_i = \frac{\sum _{j \in X_i} w_j}{\sum _{j \in X_i} 1} \end{aligned}$$

It is clear that static voxels should already have their correct value from the initial volume, which means all changes to this initial volume in the update step will be due to the dynamic process which is being studied. However, in reality these changes also contain contributions from noise in both dynamic and static regions and potentially other imaging artefacts. In contrast to the techniques discussed earlier, the static regions in the initial volume do not have to be exactly correct when non-zero weights are used, as will be discussed in more detail in the following sections. Nevertheless, it is important that the initial volume is a good approximation of the sample in the static regions of the sample, such that most of the back projected information in the update step physically originates from the dynamic regions. Typically, a high-quality reconstruction obtained from a scan before the dynamic process was initiated is used as initial volume.

The benefit of fast convergence and the utilization of a previously acquired initial volume makes the technique perfectly suited for 4D-$\mu$CT imaging. The reconstruction strategy for a 4D-$\mu$CT acquisition starts by subdividing the radiographs into a number of subsets which may overlap, preferably spanning at least a complete angular range in each subset. Each will be reconstructed separately with the technique described above and the combination of these 3D volumes forms the resulting 4D volume, i.e. all these 3D arrays are put into a 4D array together.

The choice of the weight volume has an important influence on the result, as will be discussed in the results section. In this work, we consider four different cases by combining two variables: (1) whether $w_j = 0$ is possible or not, and (2) whether the weight volume is discrete or continuous. As can be seen in Eq. (2), any voxel *j* with $w_j = 0$ will never deviate from $\mu ^0_j$. Therefore, the initial volume is not allowed to contain any inaccuracies in these voxels. In contrast, if the lowest possible $w_j$ is some value larger than 0, these static regions might still change from their initial value. The hypothesis is that the noise will have a bigger impact when redistributed over only a few dynamic voxels, thus reducing the signal-to-noise ratio. Another distinction between different weight volumes is whether they have a limited amount of different $w_j$ or if any value is possible. We will call this discrete and continuous weight volumes, respectively. It is important that the structures that are likely to change during the process can be correctly annotated. Typically, as in this work, this will be done based on a high-quality $\mu$CT scan acquired before or after the process.

In case of a discrete weight volume, the segmentation is performed with a simple threshold. In a continuous weight volume, the weights are related to the reconstructed attenuation coefficients, e.g. with a Gaussian, such that voxels with a value close to the attenuation coefficient of pores will receive a high weight:

4 $$\begin{aligned} w_j = b+v\cdot \exp \left( -\frac{(\mu _j-\mu _{centr})^2}{2\sigma _w^2} \right) \end{aligned}$$

with $\mu _j$ the attenuation coefficient in the reconstructed pore volume, $\mu _{centr}$ the average attenuation coefficient of a pore and *v* and $\sigma$ parameters that determine the height and width of the Gaussian, respectively. *b* is a constant determining a minimum weight for the static voxels, and should be set to $b = 0$ when zero weights are allowed. It is noteworthy that the exact values of the weights are not relevant, as they are normalised by Eq. (3).

It is important to note that the function that determines the weights is likely to have a significant impact on the results, and will depend on the object under investigation. However, a detailed study of this impact and potential optimizations is beyond the scope of this proof-of-concept paper.

## Results and discussion

**Figure 1:**
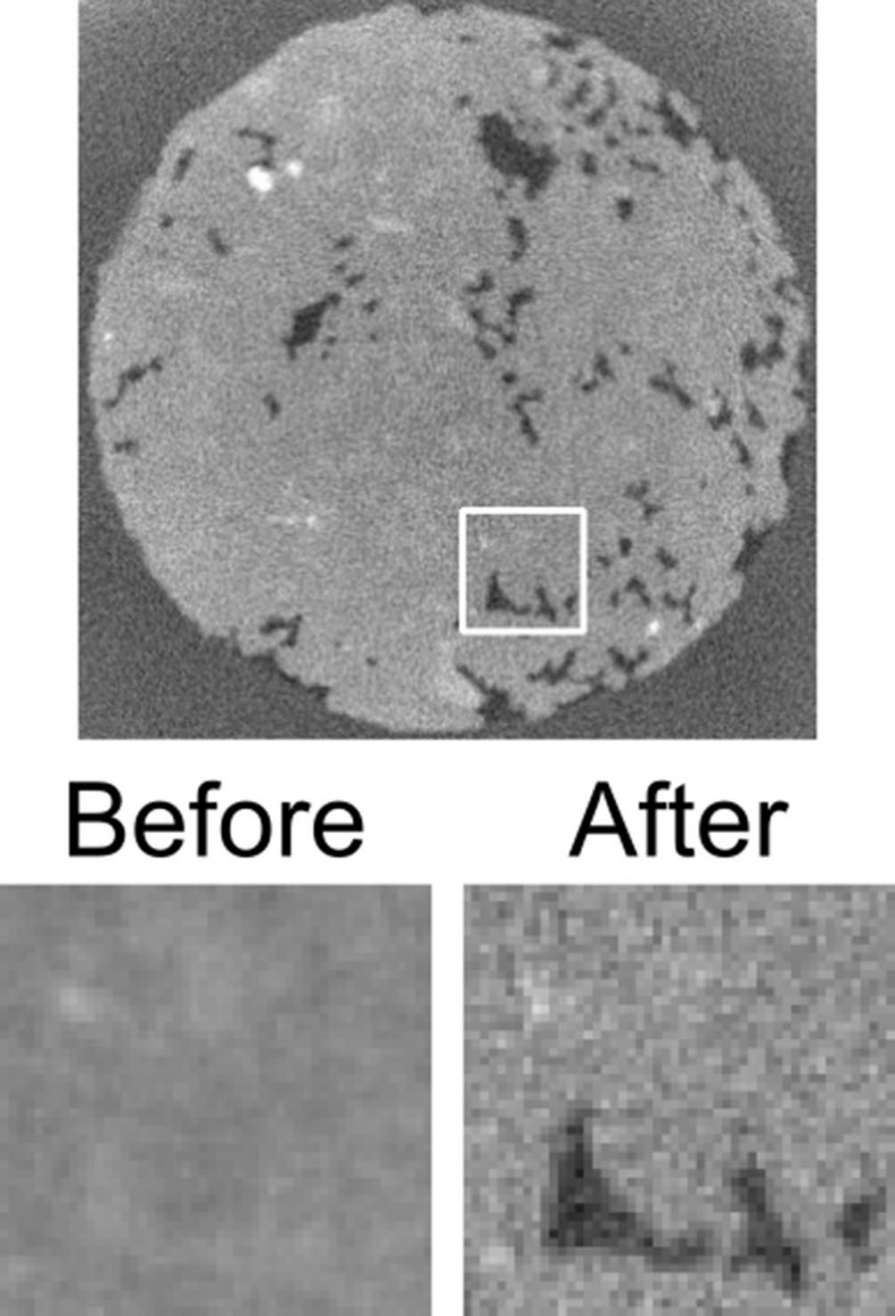
The central slice of the reference reconstruction with a zoomed-in area focusing on a specific pore shown in the bottom figures. This is an averaged reconstruction without prior knowledge using all projections. The zoomed-in area shows the situation of the pore before filling with oil and after filling with oil. The complete slice shows the after-filling situation. The diameter of the slice is 6 mm and the physical size of the zoomed-in region is $0.95\, \hbox {mm} \times 0.95\,\hbox {mm}$. Shown attenuation coefficients are linearly scaled between 0/mm (black) and 0.15/mm (white).

**Figure 2:**
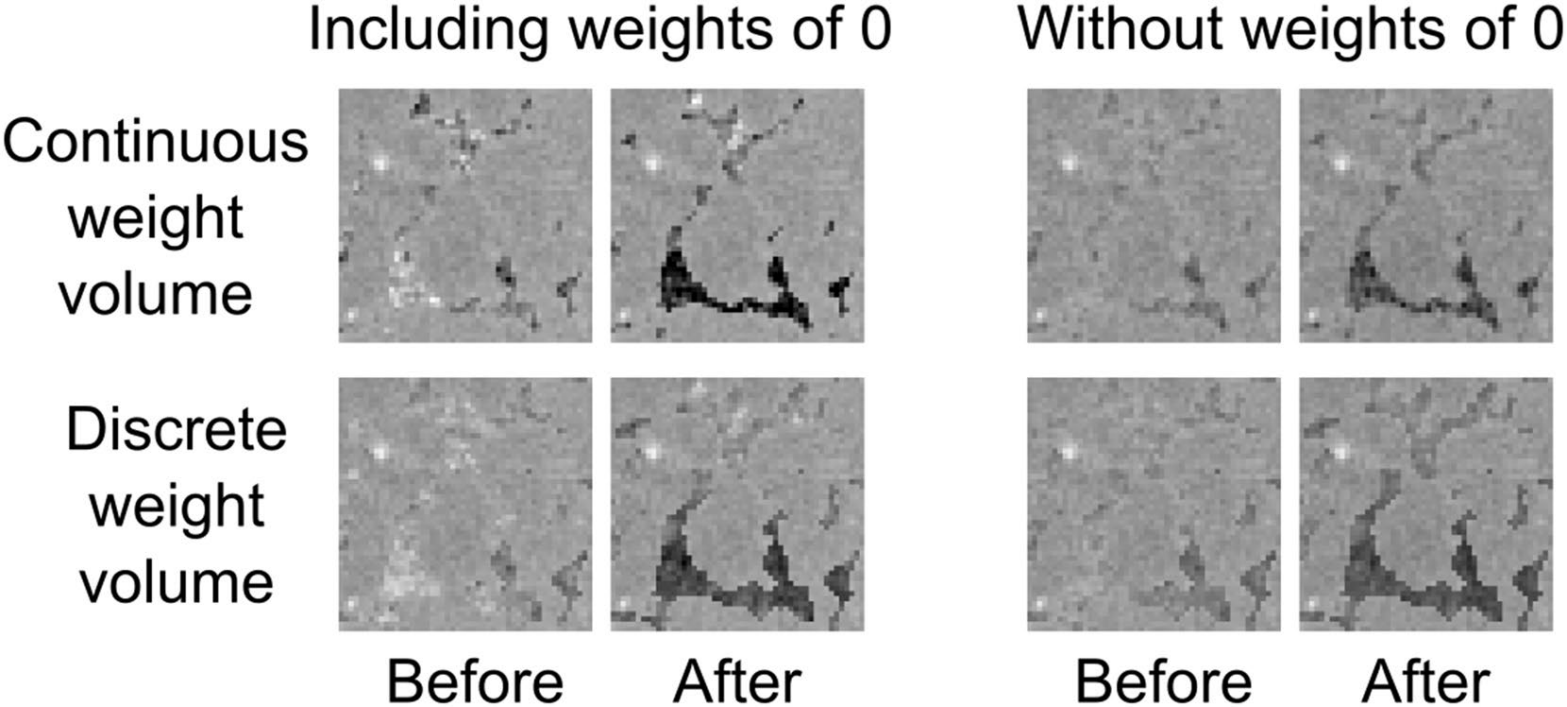
Zoom-in of the pore featured in Fig. 1 in the central slice of weighted back projection reconstructions with different weight volumes using only $1/10{\text {th}}$ of the available projections. Two reconstructed time steps are shown for each one: immediately before the filling of this pore with oil and immediately after. The physical size of the displayed region is $0.95\, \hbox {mm} \times 0.95\, \hbox {mm}$. Shown attenuation coefficients are linearly scaled between 0/mm (black) and 0.15/mm (white). The left part of the pore in the before-filling time step shows a lot of noise when including weights of 0.

**Figure 3:**
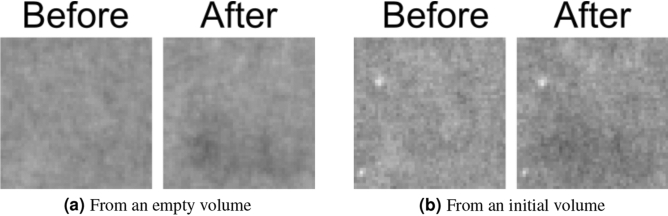
Zoom-in of the pore featured in Fig. 1 in the central slice of two different reconstructions using only $1/10{\text {th}}$ of the available projections: the conventional SART reconstruction starting from an empty volume and starting from an initial volume. Two reconstructed time steps are shown for each one: immediately before the filling of this pore with oil and immediately after. The physical size of the displayed region is $0.95\, \hbox {mm} \times 0.95\, \hbox {mm}$. Shown attenuation coefficients are linearly scaled between 0/mm (black) and 0.15/mm (white).

**Figure 4:**
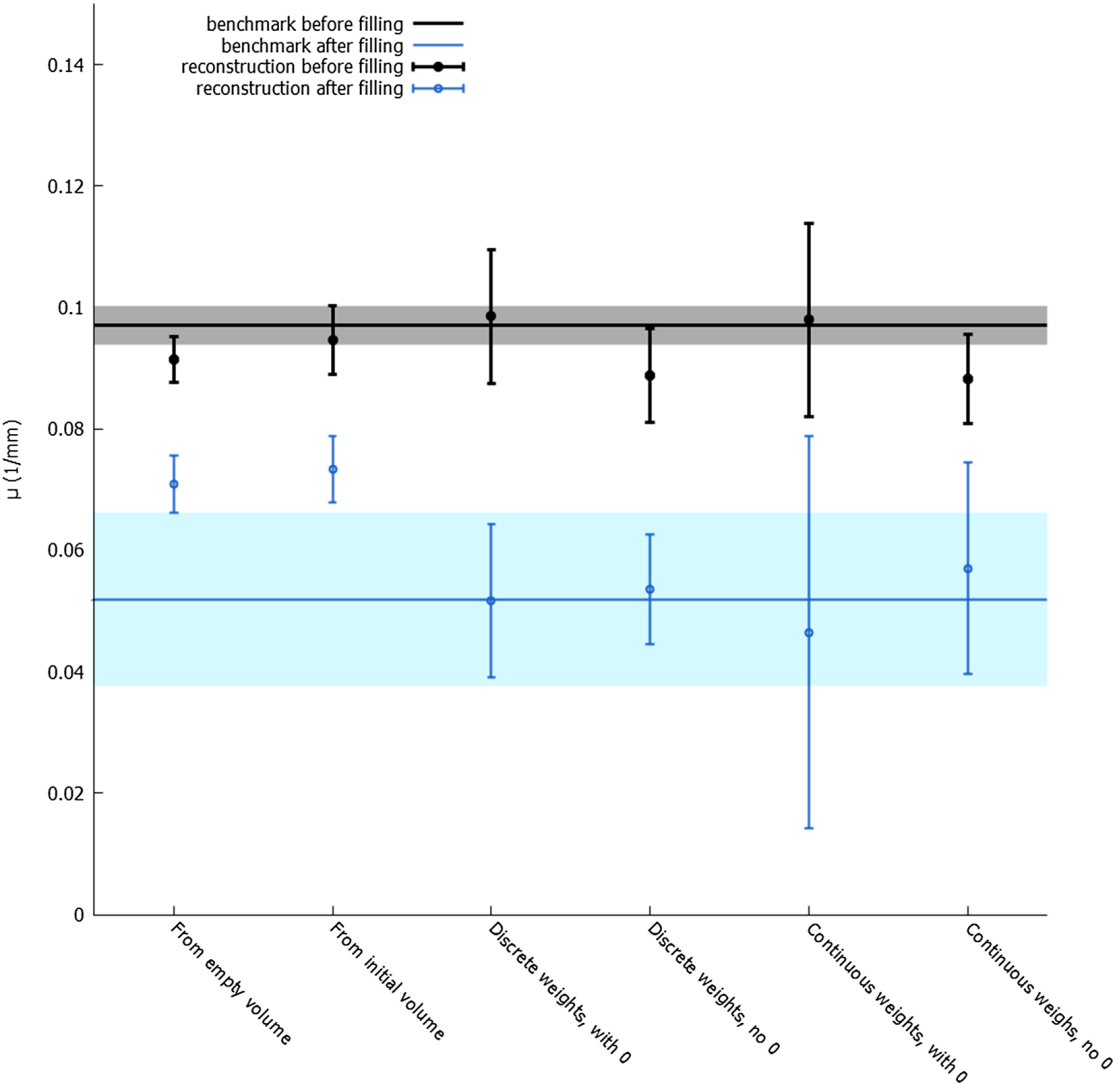
The average attenuation coefficient and average standard deviation in a dynamic pore (the bigger pore in the zoomed-in region in Fig. 2) in the reconstructions which used $1/10{\text {th}}$ of projections. Data from both before the filling with fluid and after are shown. The $\mu$ from the reference reconstruction are shown as a constant function, with the $\sigma$ on the reference reconstruction denoted by the lightly coloured areas around the constant function.

### Influence on dynamic regions

The experimental data used to evaluate the method is described in detail in the Materials & Methods section, and consists out of an in-situ 4D-$\mu$CT scan of oil displacing brine in the pores of a rock sample. As we are most interested in applying the method to fast imaging experiments with limited angular sampling, each reconstructed time step in the dynamic scan consists out of only 60 projection images equally spaced over a $360^{\circ }$ acquisition. The dynamic process in this experiment only occurs in the pores. The weight volume should thus contain high values for the pore voxels and low values for the other voxels. The pore structure and the initial fluid filling are determined from high-quality pre-scans of the sample.

In this section, we will describe the results using each of the described types of weight volumes and evaluate these based on the high-quality reference reconstruction, which is shown in Fig. 1. The result of the weighted back projection reconstructions with different weight volumes are shown in Fig. 2, where two points in time are shown for each reconstruction, i.e. just before and after the moment this specific pore was invaded by oil (the Haines jump^12^).

The slices in Fig. 3 show that both the reconstruction from an empty and from an initial volume, i.e. the reconstructions that did not use a weight volume, fail completely at reconstructing the feature of interest correctly. The quantitative analysis (Fig. 4) shows that the normal SART reconstruction and the reconstruction from an initial volume fail to reach the attenuation coefficient of the reference volume after filling of the pore with oil. It is clear that the number of projections in this limited dataset is far too few. In contrast, the weighted back projection reconstructions in Figs. 2 and 4 perform well, clearly showing the pore getting filled for all four weight volumes and the attenuation coefficients being close those of the reference reconstruction. From visual assessment, there is little difference between discrete or continuous weight volume reconstructions. The attenuation coefficients in Fig. 4 are similar, although the noise for a continuous weight volume is found to be larger. The difference between including or excluding $w_j = 0$ is larger: the noise is stronger when including $w_j = 0$, as shown in the size of the error bars. This noise is clearly visible when visualizing a single slice as function of time (see supplementary materials): many smaller pores are ‘flickering’ around the attenuation coefficient of the surrounding rock. This is almost certainly not a physical effect but an artefact caused by the method. Using piecewise linear fitting^27^, a method for temporal smoothing and analysis, these small pores were found to be static and should therefore remain on their initial attenuation coefficients. This strong noise behaviour is much more subdued in the weighted back projection reconstructions that use a minimal weight larger than 0.

The increased noise susceptibility is caused by the nature of weighted back projection: the pores, which have a higher weight than the surrounding rock, receive the high amount of noise that would otherwise be spread out over a much larger length. Indeed, in the case of conventional reconstructions, this is the complete length of the reconstructed volume with an equivalent contribution for each voxel. For weighted back projection, the length is primarily (and even exclusively in the case where zero weights are allowed) determined by the pore space, which is only a small fraction of the volume. When zero weights are allowed, the spread-out length is shortest, explaining why the noise in the pores is the most pronounced in these reconstructions.

This higher noise in the dynamic regions is the largest disadvantage of the weighted back projection technique. It is stronger when zero weights are allowed, since the static regions in that case cannot act as a buffer for some of the noise present in the back projected data. At the other hand, not only the propagation of noise is stronger in weighted back projection, but also of the signal itself. This is why the filled pores are clearly visible in the weighted back projection reconstructions even when using only a limited amount of projections.

**Figure 5:**
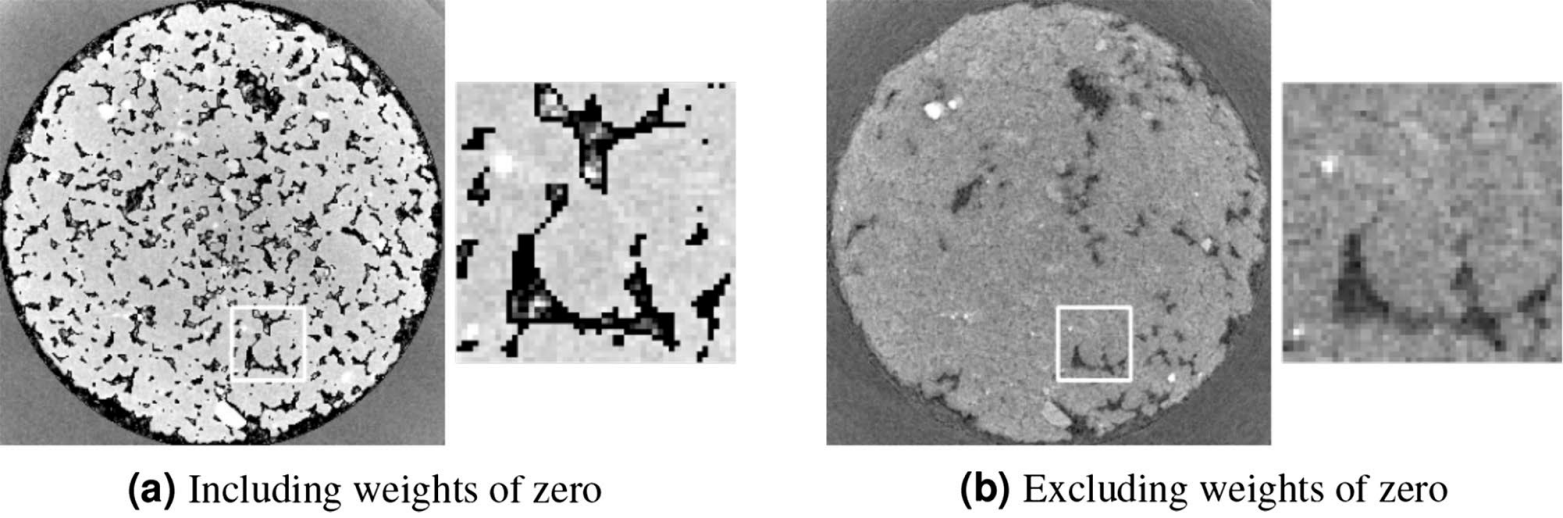
Weighted back projection reconstructions starting from a wrong initial volume using a weight volume where $w_j = 0$ is possible (Fig. 5a) and another where $w_j > 0$ for all voxels *j* (Fig. 5b). These reconstructions used all available projections, i.e. 600 (the full amount in one rotation) per time step. The shown time step is after the filling of the chosen pore with oil. All shown slices and zoom-ins are scaled equally, between 0/mm (black) and 0.15/mm (white). A reference reconstruction for comparison can be found in Fig. 1.

### Effect of errors in prior knowledge

In general, reconstruction methods which incorporate prior knowledge are highly susceptible to errors in this prior knowledge. In this case, weighted back projection methods with zero-weights, can be expected to result in strongly deteriorated reconstruction quality when the static regions of the initial volume, i.e. the voxels where $w_j = 0$, are wrong. To evaluate this, we consider an initial volume in which the attenuation coefficients are about 33% higher than they are in the reconstructions of the dynamic scan. This error can be caused by a multitude of effects, and is likely to go unnoticed hence should be correctly coped with in the reconstruction.

As shown in Fig. 5, allowing zero weights in this case renders the reconstruction useless: the dynamic pores at the bottom of the zoomed-in region are shown as filled, but so is every other pore in the volume, including those that should have remained on their initial value. On the other hand, when the static regions still have a low, non-zero weight, the reconstruction can handle a small error in the prior knowledge.

From this, we can conclude that weight volumes without zero weights, both discrete or continuous, are more robust to small errors in the initial volume. It is noteworthy that the weight volumes used for this work are obtained by manually setting a threshold and a mapping function, and further work is necessary to define (preferably automated) methods to optimize these volumes. Zero weights, while useful in theory, produce too much noise to safely discern the real change to the initial volume, even when using a high-fidelity initial volume.

**Figure 6:**
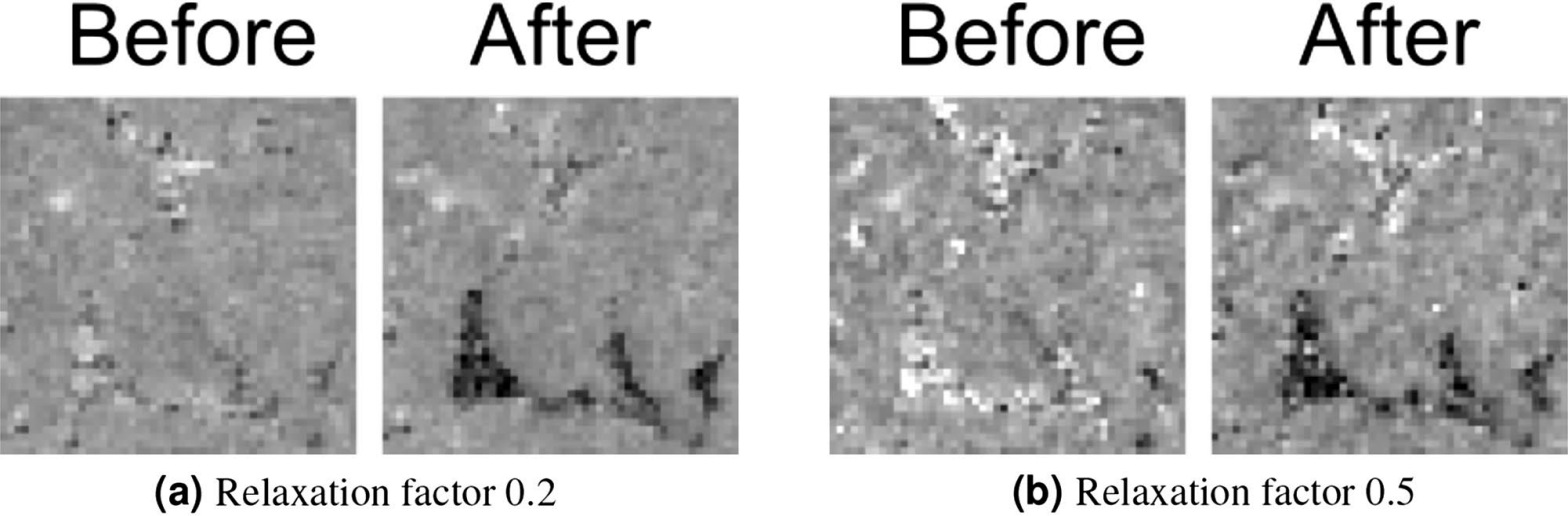
Slices of a weighted back projection reconstruction with two different relaxation factors $\alpha$. Shown attenuation coefficients are linearly scaled between 0/mm (black) and 0.15/mm (white).

### Relaxation factor

**Figure 7:**
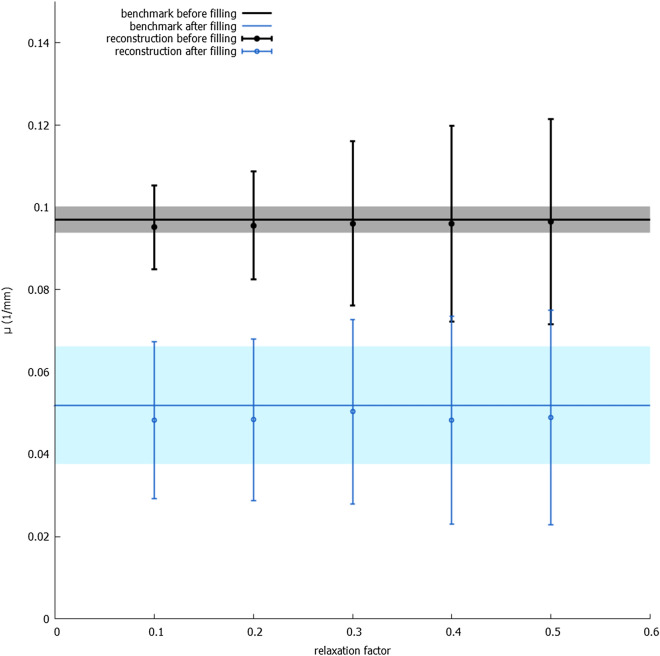
The average attenuation coefficient and average standard deviation in a dynamic pore (the bigger pore in the zoomed-in region in Fig. 2) in the reconstructions which used all available projections, using different relaxation factors. Data from both before the filling with fluid and after are shown. The $\mu$ from the reference reconstruction are shown as a constant function, with the $\sigma$ on the reference reconstruction denoted by the lightly coloured areas around the constant function.

The relaxation factor $\alpha$, as used in Eqs. (1) and (2), is an important consideration in weighted back projection. The reconstructions shown in the previous paragraph had $\alpha = 0.5$ for the normal reconstruction and the classical initial volume reconstruction, but $\alpha = 0.2$ for the weighted back projections. These values were chosen to take the high susceptibility to noise of the weighted back projection, mentioned earlier, into account. It is known that decreasing the relaxation factor results in a lower noise level. This is visible in Fig. 6: a high relaxation factor in weighted back projection results in high noise in the pores, i.e. in the regions with high weights. However, simply lowering the relaxation factor already decreases this noise. The same is visible in Fig. 7: a lower relaxation factor lowers the standard deviation $\sigma$.

A disadvantage of a lower relaxation factor, when considering a normal reconstruction, is the smoothing it causes^28^. However, because of the different weights in different areas, this smoothing effect is not visible in the weighted back projection reconstruction. We conclude that, in order to get the same noise and smoothing behaviour, the relaxation factor should be lower for a weighted back projection and higher for a normal reconstruction.

### Computational considerations

The practical implementation of the method introduces an additional volume in the reconstruction process. Particularly for GPU implementations, memory requirements should be kept as low as reasonably achievable. For this work, a volume with the same amount and configuration of voxels as the reconstruction volume was used, yet at a lower precision. More specifically, only 8-bit integers were used (using a fixed scaling factor to obtain non-integer weights) to reduce memory requirements. Alternatively, a lower resolution weight volume can be implemented, using interpolation to obtain the weights of each voxel in the reconstruction grid. This approach was not applied here to avoid influence of the interpolation step. Furthermore, this method increases the computational complexity, resulting in a trade-off between memory requirements and computational speed. Furthermore, such methods will reduce spatial resolution, which is an undesired effect.

## Conclusions

We presented a new technique to include prior knowledge on dynamic and static regions directly into an iterative reconstruction of 4D-$\mu$CT data. This technique uses an additional volume, the weight volume, which outlines the regions in the sample that are more likely to undergo changes due to the dynamic process. In order to use this information, an initial volume is also required.

An important class of research problems is dynamic processes occurring within a static grid, which is where weighted back projection is applicable. An example of such a process is fluid flow through the pores of a geomaterial^12^. In contrast, weighted back projection is less suited for dynamic processes which consist mainly of deformation, such as the deformation of foams^8^, since in those cases no static regions can be identified and prior knowledge on locations in the foam can not be used as a 1-to-1 mapping with future locations. As a future prospect, weighted back projection could be combined with motion registration^29–31^, deforming not only the volume itself but the weight volume with it, to include more possible applications.

The addition of weights in the back projection allows the reconstruction to be successful using a smaller input dataset, i.e. fewer projections, while still enabling to accurately detect changes in the volume. This means the $\mu$CT scanner could rotate faster and therefore the time resolution of the reconstruction will improve. Note that decreasing the number of projections in a continuous scan means that each projection spans a larger angle, thereby increasing the angular smoothing. As this will affect the final image quality, notably the spatial resolution, there will be a minimal number of necessary projections to achieve a sufficient image quality. The latter is a qualitative term as it depends strongly on the requirements imposed by morphology, contrast and post-processing methodology of the given application. The proposed method is also shown to exhibit sharpening, as the spatial resolution from the high-quality scan used to create the weight volume is propagated to the reconstructions. Additionally, weighted back projection is more likely to suffer from noise in comparison to a regular reconstruction, since the noise is back projected over a smaller region. However, this can be compensated by using a lower relaxation factor, as demonstrated in this work.

This study compared different possible weight volumes and their effect on the resulting reconstruction. While the difference between a continuous and a discrete weight volume was not large, it is clear that the static region preferably has a value higher than zero. This allows for the noise to ‘dissipate’ in the large static region and, more importantly, leaves room for error correction in the initial volume, which is an important quality for a reconstruction algorithm using prior knowledge.

The proposed methodology is not limited to the implementation and application presented in this manuscript. Given the generic nature of this method, it can be combined with a large number of implementations and optimizations of iterative reconstruction algorithms. While very interesting, an extensive quantitative study using a broad range of simulated datasets is beyond the scope of this proof-of-principle study, focusing on qualitative evaluation of a real dataset, in which we’ve shown the advantages and the caveats of the method. We believe the method can become a fundamental building block for reconstruction algorithms and has the potential to significantly enhance data analysis in specific cases.

## Materials and methods

### Data acquisition

The methods investigated in this paper are demonstrated on a 4D-$\mu$CT dataset obtained to study the behaviour of fluids in the pores of porous geomaterials, such as groundwater flow in rocks and sediments. The dynamic process studied in this dataset was drainage of a Bentheimer sandstone, during which oil intruded a water-filled porous rock. This is of importance to the study of groundwater pollution, petroleum reservoirs and ${\hbox {CO}}_2$ sequestration^12,14,32,33^.

The sandstone sample was roughly cylindrical, with a height of 10 mm and a diameter of 6 mm. The experimental data from this sandstone was acquired using the gantry-based EMCT $\mu$CT scanner^34^ of the UGent Centre for X-ray Tomography (UGCT) as described in detail by Bultreys et al.^14^. In total, three separate $\mu$CT datasets were acquired. The first scan was a high quality static scan of the rock in which the pores were filled with water. This scan took 17 min and 52 sec and consisted of 2201 projections of 487 ms exposure each, obtained in one full rotation. This resulted in a volume in which the pores were clearly visible, which we use as a basis for the weight volume. The second scan was a high quality static scan of the rock, but now the pores were filled with an aqueous CsCl solution (10 wt% CsCl), made to match the attenuation coefficient of the rock’s solid quartz grains. As such, the dynamic process of oil intrusion is imaged as a binary volume with only two attenuation coefficient values. Over 14 min and 38 sec, 1801 projections, also of 487 ms exposure each, were taken, covering one full rotation. The reconstruction of this scan will be used as the initial volume. Both datasets were acquired at full detector resolution (1316x1312 pixels) but are reconstructed on a $512 \times 512 \times 512$ voxels grid. Both these scans were performed with more time for one rotation compared to the third, dynamic scan described next, so they could be reconstructed with a higher signal-to-noise ratio.

Finally, a fast dynamic scan was taken of the rock, starting from the situation where the pores were filled with the aqueous CsCl solution, while oil (n-decane) was pumped in from below at 0.005 ml/min. This scan took 12.86 sec per rotation and covered 79.8 rotations during which 48,000 projections of 658x656 pixels were acquired, i.e. 600 per full rotation (not including dark images and flat fields for normalisation). This is a time-resolved scan, meaning that this is a continuous sequence of multiple rotations, and full-rotation reconstructions can be made at arbitrary points in time.

### Software implementation , reconstruction parameters and datasets

The proposed method is implemented in CTrex, an in-house developed platform for GPU-based iterative reconstructions^35^. In this implementation, Joseph’s method^36^ is used to approximate $s_{ij}$ in Eq. (1). It is run on a powerful personal desktop computer with an AMC Ryzen 5 2400 G CPU and 64 GB RAM containing an NVIDIA GeForce GTX 1080 Ti GPU (11 GB memory).

All reconstructions in this manuscript used one full iteration of the SART algorithm, i.e. used every projection once. We compared the reconstructions with a reference volume, for which we reconstructed two locally static situations, representing an average over the first 8 rotations and the last 8 rotations of the dynamic scan. The projections corresponding to these 8 rotations were averaged and the resulting dataset was reconstructed with conventional SART. Due to small numerical deviations in the encoder of the rotation stage, the projections of each rotation were shifted $0.096^{\circ }$ compared to the previous rotation, which resulted in angular smoothing when averaging over many rotations. The number 8 was chosen to get significant noise reduction while keeping the angular smoothing limited (i.e. similar to the angular interval between two projection images). The pore on which the figures zoom in was static in both reference reconstructions and got filled with oil in between.

**Figure 8:**
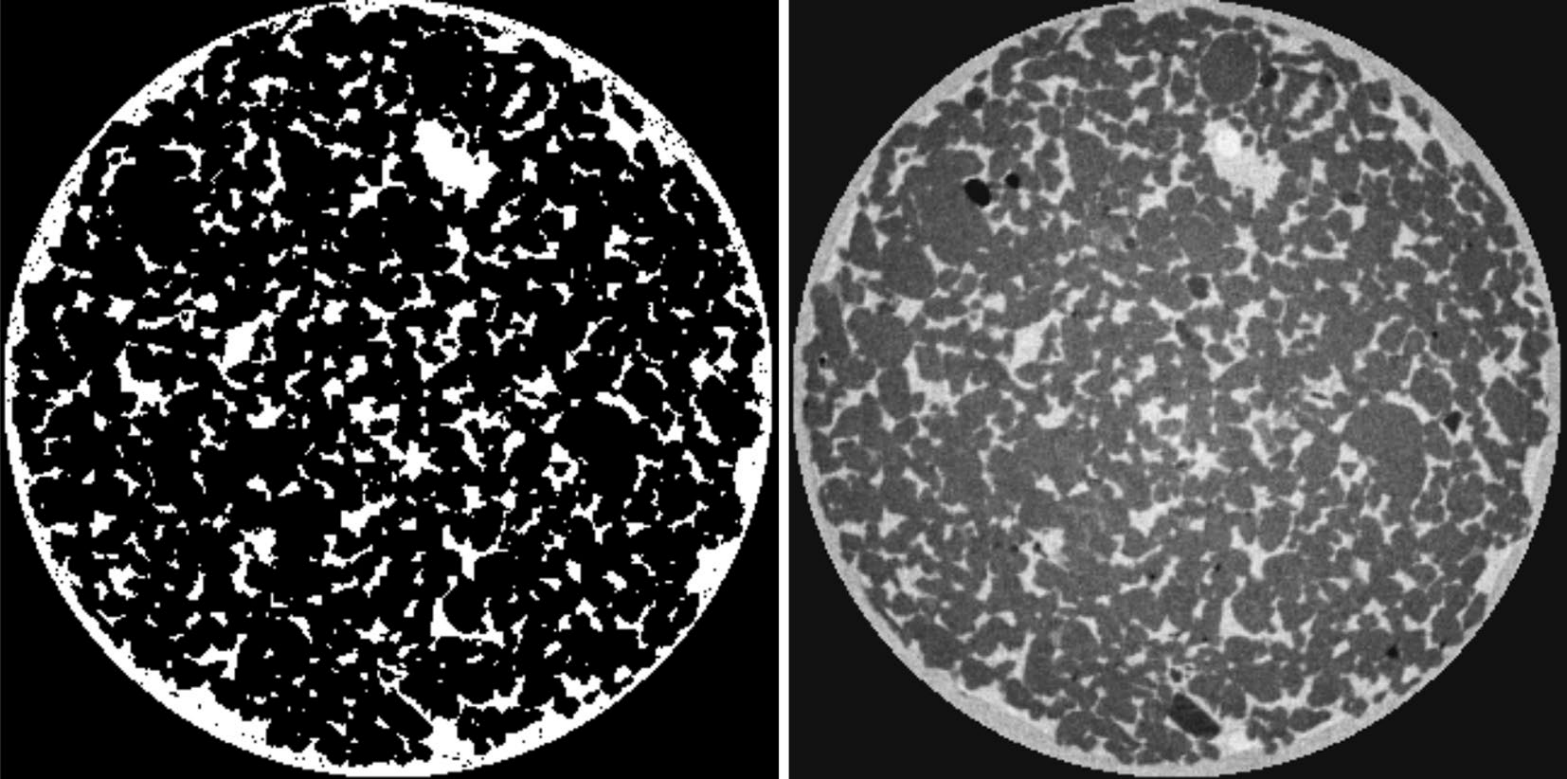
The central slice of a discrete (left) and a continuous (right) weight volume with zero weights allowed for the Bentheimer sandstone. The images are scaled linearly between 0 and 1.

The weight volumes were derived from an initial high-quality scan where the pores were filled with water such that all pores could be easily segmented. This scan was reconstructed using conventional SART. A cylindrical mask that encompassed the rock as close as possible was applied to this volume. The weights of all voxels outside of the rock were set to the lowest value, depending on the settings of the weight volume. Inside this cylinder, the pores were segmented based on their attenuation coefficients in the reconstruction. To determine the discrete weight volume, the threshold was chosen manually (see histogram in Supplementary Materials). The weights are set to either $w_j = 0$ and $w_j = 20$ or $w_j = 1$ and $w_j = 20$. For the continuous weight volume, we used Eq. (4) with parameters $\mu _{centr} = 0$ /mm $\sigma _w = 0.03$ /mm, $b=0 (1)$ and $\nu = 20 (19)$ depending on the possibility of zero weights. A slice of a discrete and a continuous weight volume is shown in Fig. 8.

The same volume was used to enable a quantitative analysis by investigating the average value within a single pore. As the pore filling happened in a very short time, and the reference datasets were chosen to be sufficiently far away from this time, the attenuation coefficient of all voxels within this pore should be identical in all reconstructions. This enabled to quantify the noise level in the data for different reconstructions. The pores were segmented and labeled using the 3D analysis software Octopus Analysis (formerly known as Morpho+^37^). A large pore (2292 voxels) was selected, and for each reconstruction the average $\mu$ and standard deviation $\sigma$ of the attenuation coefficient over this volume was calculated.

The reconstructions with which the weighted back projection was evaluated used only 1 out of every 10 projections. One time step was therefore reconstructed with 60 projections, uniformly spread over a full rotation. This simulates a dynamic process that is ten times faster with a 4D-$\mu$CT scan that is performed 10 times faster. The reconstruction quality of this dataset, with and without using an initial volume, is shown in Fig. 3b.

Since the reference reconstruction (Fig. 1) uses the full available data, we can compare such reconstructions of such a ‘fast’ scan with limited available data with a higher quality scan.

## Supplementary information

Supplementary Information 1.

Supplementary Information 2.

Supplementary Information 3.

Supplementary Information 4.

Supplementary Information 5.

Supplementary Information 6.

Supplementary Information 7.

## Data Availability

The datasets generated during and/or analysed during the current study are available from the corresponding author on reasonable request.
