# Supplementary figures and images for: Improving image quality in fast, time-resolved micro-CT by weighted back projection
Source: Sci Rep. 2020 Oct 22;10:18029. doi: 10.1038/s41598-020-74827-x (PMC7581769; doi:10.1038/s41598-020-74827-x)

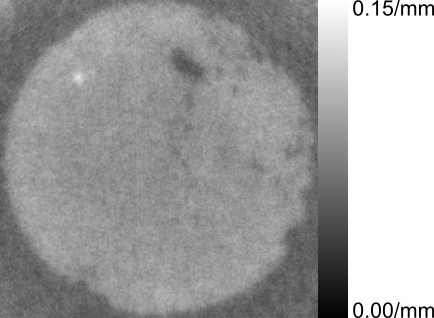

Supplement: Supplementary file 1 — Supplementary Information 1. [file 41598_2020_74827_MOESM1_ESM.gif]

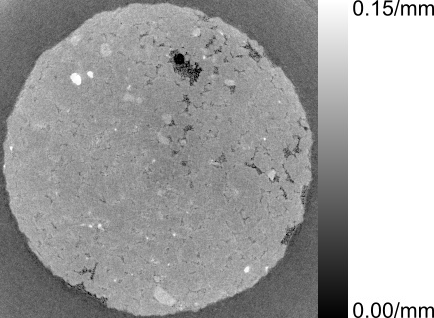

Supplement: Supplementary file 3 — Supplementary Information 3. [file 41598_2020_74827_MOESM3_ESM.gif]

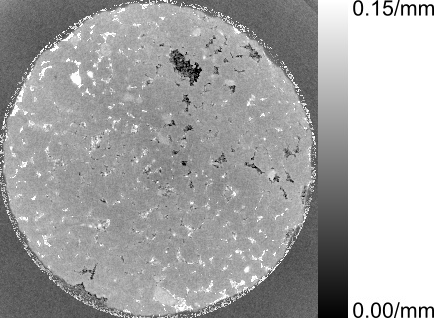

Supplement: Supplementary file 4 — Supplementary Information 4. [file 41598_2020_74827_MOESM4_ESM.gif]

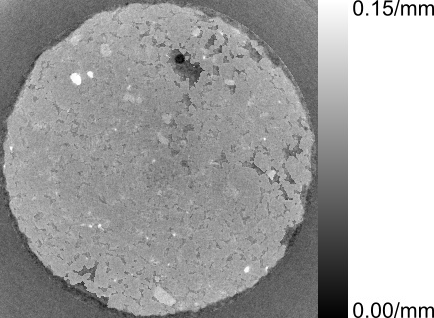

Supplement: Supplementary file 5 — Supplementary Information 5. [file 41598_2020_74827_MOESM5_ESM.gif]

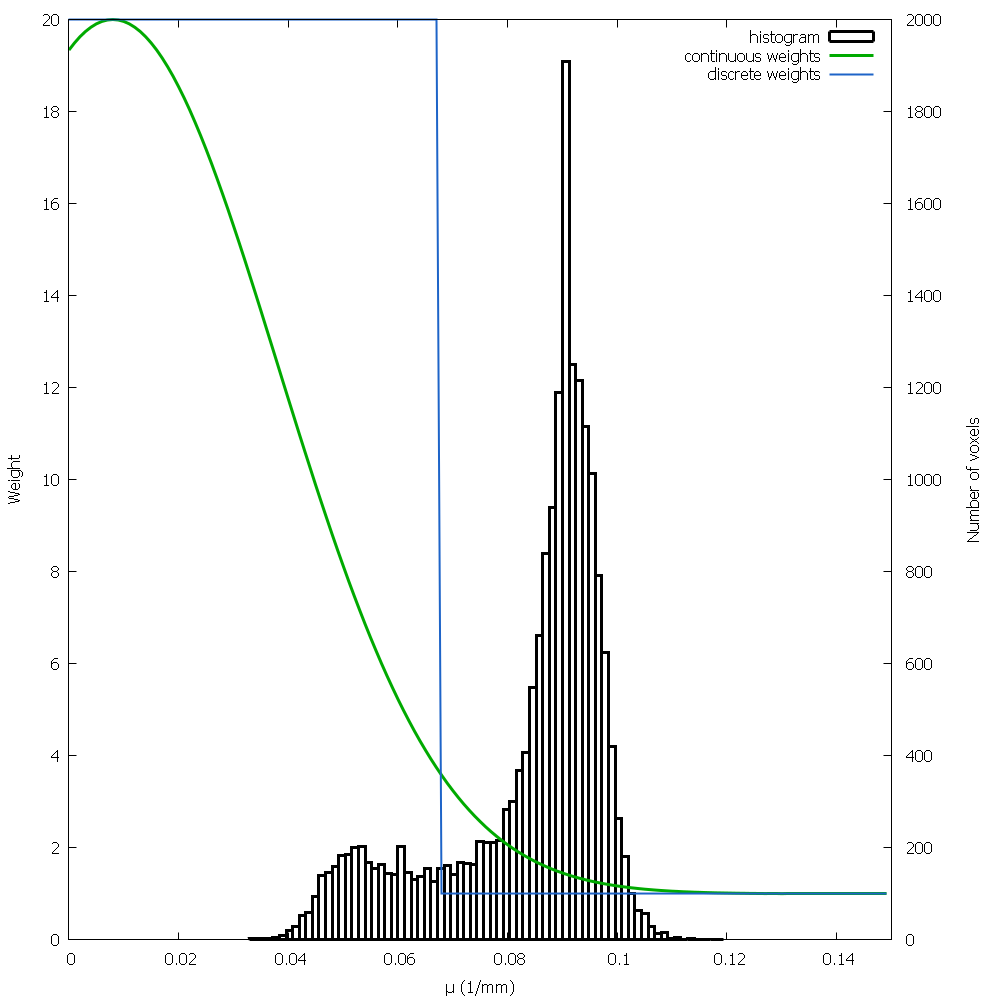

Supplement: Supplementary file 7 — Supplementary Information 7. [file 41598_2020_74827_MOESM7_ESM.png]
