# Supplementary material for: Improving image quality in fast, time-resolved micro-CT by weighted back projection
Source: Sci Rep. 2020 Oct 22;10:18029. doi: 10.1038/s41598-020-74827-x (PMC7581769; doi:10.1038/s41598-020-74827-x)
Supplement: Supplementary file 2 — Supplementary Information 2. [file 41598_2020_74827_MOESM2_ESM.pdf]

Improving image quality in fast, time-resolved micro-CT by weighted back projection

Marjolein Heyndrickx[1], Tom Bultreys[2], Wannes Goethals[1], Luc Van Hoorebeke[1], and Matthieu N. Boone[1,*]

[1]UGCT / Radiation Physics, Dept. Physics and Astronomy, Ghent University, Proeftuinstraat 86, N12, 9000 Ghent, Belgium
[2]UGCT / PProgRess, Dept. Geology, Ghent University, Krijgslaan 281 - Building S8, 9000 Ghent, Belgium
[*]matthieu.boone@ugent.be

ABSTRACT

Time-resolved micro-CT is an increasingly powerful technique for studying dynamic processes in materials and structures. However, it is still difficult to study very fast processes with this technique, since fast scanning is typically associated with high image noise levels. We present weighted back projection, a technique applicable in iterative reconstruction methods using two types of prior knowledge: 1) a virtual starting volume resembling the sample, for example obtained from a scan before the dynamic process was initiated, and 2) knowledge on which regions in the sample are more likely to undergo the dynamic process. Therefore, processes on which this technique is applicable are preferably occurring within a static grid. Weighted back projection has the ability to handle small errors in the prior knowledge, while similar 4D micro-CT techniques require the prior knowledge to be exactly correct. It incorporates the prior knowledge within the reconstruction by using a weight volume, representing for each voxel its probability of undergoing the dynamic process. Qualitative analysis on a sparse subset of projection data from a real micro-CT experiment indicates that this method requires significantly fewer projection angles to converge to a correct volume. This can lead to an improved temporal resolution.
